# Ectopic Cushing Syndrome Secondary to Corticotropin-secreting Wilms Tumor: A Rare Paraneoplastic Phenomenon

**DOI:** 10.1210/jcemcr/luaf325

**Published:** 2026-04-21

**Authors:** Sonali Palai, Bijay Kumar Sahoo, Shamli Mishra, Satyaprada Mishra, Sudhiranjan Pattanaik, Debarchan Jena

**Affiliations:** Department of Endocrinology, S.C.B. Medical College, Cuttack, Odisha 753007, India; Department of Endocrinology, S.C.B. Medical College, Cuttack, Odisha 753007, India; Department of Endocrinology, S.C.B. Medical College, Cuttack, Odisha 753007, India; Department of Endocrinology, S.C.B. Medical College, Cuttack, Odisha 753007, India; Department of Endocrinology, S.C.B. Medical College, Cuttack, Odisha 753007, India; Department of Endocrinology, S.C.B. Medical College, Cuttack, Odisha 753007, India

**Keywords:** ectopic ACTH secretion (EAS), nephroblastoma, paraneoplastic, ACTH immunohistochemistry

## Abstract

Ectopic Cushing syndrome (ECS) is an exceptionally rare cause of endogenous hypercortisolism in children, accounting for less than 1% of pediatric Cushing syndrome (CS) cases. We report a rare case of ECS in an 8-year-old girl secondary to an adrenocorticotrophic hormone (ACTH)-secreting Wilms tumor. She exhibited classical features of hypercortisolism, including rapid weight gain, hypertension, hyperpigmentation, easy bruisability, and a palpable abdominal mass. Laboratory evaluation revealed markedly elevated serum cortisol and ACTH levels, with absent cortisol suppression following low- and high-dose dexamethasone suppression tests, suggestive of ectopic ACTH secretion. Pituitary and thoracic imaging were unremarkable. Contrast-enhanced abdominal computed tomography identified a large left renal mass. She underwent left radical nephrectomy with perioperative hydrocortisone supplementation. Postoperatively, she showed rapid clinical improvement with a significant decline in ACTH levels. Histopathology confirmed a stage III triphasic Wilms tumor with favorable histology. Although initial ACTH immunostaining was negative, repeat staining demonstrated focal ACTH positivity, suggesting tumor heterogeneity. She was subsequently treated with chemotherapy and radiotherapy. Persistently low cortisol levels postsurgery indicated hypothalamic-pituitary-adrenal axis suppression, requiring continued glucocorticoid replacement. This case underlines the importance of considering ectopic ACTH-producing tumors in pediatric Cushing syndrome and highlights the diagnostic complexities associated with focal hormone expression.

## Introduction

Cushing syndrome (CS) is a complex endocrine disorder resulting from prolonged glucocorticoid excess, either from exogenous administration or endogenous overproduction. In children, exogenous CS remains the predominant form because of widespread therapeutic glucocorticoid use, whereas endogenous CS accounts for only ∼10% of cases [1]. Among endogenous causes, ACTH-dependent CS is most frequently observed in children older than age 5 years, with pituitary corticotroph adenomas (Cushing disease) comprising nearly 75% to 80% of cases. In contrast, adrenocortical carcinoma represents the predominant cause in children younger than age 5 years [2].

Ectopic Cushing syndrome (ECS), resulting from nonpituitary ACTH-producing tumors, is exceedingly rare. A systematic review of 161 pediatric cases showed bronchial (28%) and thymic (23%) neuroendocrine tumors as the most common causes, followed by primitive cell-derived (19%) and gastro-entero-pancreatic tumors (14%). Primitive cell-derived tumors were more common in those aged ≤10 years, whereas bronchial lesions predominated in adolescents [3].

Ectopic ACTH secretion (EAS) in Wilms tumor is extremely rare, with only a few reported cases despite Wilms tumor comprising 5% to 10% of pediatric solid malignancies [4-11]. We describe a rare case of ECS secondary to an ACTH-secreting Wilms tumor, highlighting diagnostic challenges and the need to consider paraneoplastic hormone production in pediatric cancers.

## Case Presentation

**Figure 1.**
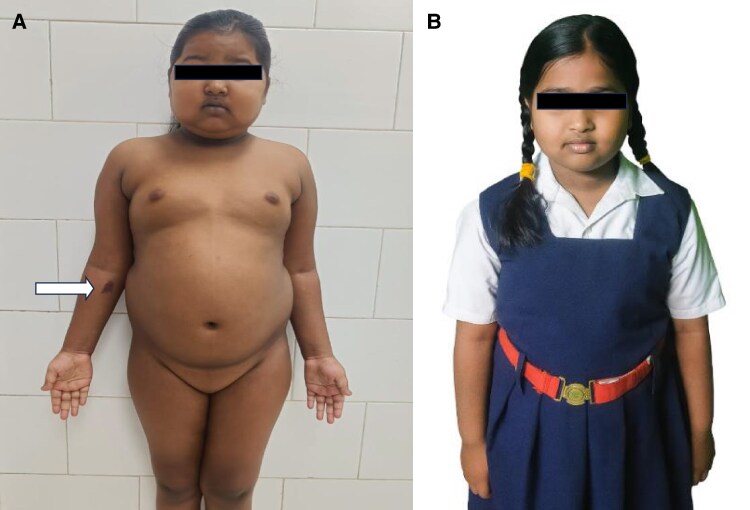
Clinical images of an 8-year-old girl with ectopic Cushing syndrome resulting from an ACTH-secreting Wilms tumor. (A) Preoperative image showing cushingoid facies, generalized weight gain, hyperpigmentation, easy bruisability (white arrow), and protuberant abdomen. (B) Follow-up image taken 3 months after tumor resection, showing resolution of cushingoid features with significant reduction in facial fullness and body weight.

An 8-year-old girl presented with a 4-month history of rapid weight gain, facial puffiness, hyperpigmentation, and increased abdominal girth. There was no history of exogenous steroid use. Examination showed cushingoid facies, dorsocervical fat pad, hyperpigmentation of skin and buccal mucosa, easy bruisability, grade 4 acanthosis nigricans, and bilateral pitting pedal edema, but no proximal myopathy (Fig. 1A). Her weight was 37 kg (90th-97th percentile) and height was 129 cm (50th-75th percentile), with mid-parental height 154.5 cm, target height 150.2 to 160.2 cm, and projected height 161.96 cm, indicating preserved linear growth. Blood pressure was markedly elevated at 140/92 mm Hg (>99th percentile), consistent with stage 2 hypertension per the 2017 American Academy of Pediatrics Clinical Practice Guidelines. Abdominal examination revealed a firm, nontender, ballotable ∼10 × 10 cm mass in the left upper quadrant.

## Diagnostic Assessment

Initial biochemical assessment showed serum potassium of 3.3 mEq/L (3.3 mmol/L) (reference range, 3.5-5.0 mEq/L [3.5-5.0 mmol/L]), fasting plasma glucose of 75 mg/dL (4.17 mmol/L) (reference range, 65-100 mg/dL [3.61-5.56 mmol/L]), and postprandial plasma glucose of 105 mg/dL (5.83 mmol/L) (reference range, <140 mg/dL [<7.78 mmol/L]). Morning (0800 hours) serum cortisol was markedly elevated at 63.06 µg/dL (1740.17 nmol/L) (reference range, 5-25 µg/dL [137.95-689.75 nmol/L]). An overnight dexamethasone suppression test (dexamethasone 15 mcg/kg administered at 2300 hours) failed to suppress cortisol levels, with a posttest cortisol of 59.78 µg/dL (1649.25 nmol/L) (reference range, <1.8 µg/dL [50 nmol/L]). Similarly, the 2-day low-dose dexamethasone suppression test (dexamethasone 30 mcg/kg/day in 4 divided doses for 2 consecutive days) showed no suppression, with cortisol remaining elevated at 54 µg/dL (1489.86 nmol/L) (reference range, <1.8 µg/dL [50 nmol/L]). Plasma ACTH was markedly increased at 650 pg/mL (143.13 pmol/L) (reference range, 7.2-63.6 pg/mL [1.59-14.01 pmol/L]), confirming an ACTH-dependent etiology. A 2-day high-dose dexamethasone suppression test (dexamethasone 120 mcg/kg/day in 4 divided doses for 2 consecutive days) also failed to suppress cortisol, with posttest cortisol measuring 45.5 µg/dL (1255.35 nmol/L) compared with a baseline of 57.12 µg/dL (1576.89 nmol/L)—a reduction of less than 50%—thereby supporting the diagnosis of ectopic ACTH secretion (Table 1).

**Table 1.** Hormonal and clinical parameters before and after tumor resection

Parameter	Preoperative value	Postoperative day 1	Postoperative day 30	Reference range
Weight (in kg)	37	—	32	—
BP (in mm Hg)	146/100	120/90	110/70	Age-specific pediatric norms
Morning serum cortisol (8 Am)	63.06 µg/dL (1740.17 nmol/L)	—	<0.5 µg/dL (13.8 nmol/L)	5-25 µg/dL (137.95-689.75 nmol/L)
Plasma ACTH	650 pg/mL (143.13 pmol/L)	9.37 pg/mL (2.06 pmol/L)	5.50 pg/mL (1.21 pmol/L)	7.2-63.6 pg/mL (1.59-14.01 pmol/L)
Post-ONDST serum cortisol	59.78 µg/dL (1649.25 nmol/L)	—	—	<1.8 µg/dL (<50 nmol/L)
After 2-day LDDST serum cortisol	54 µg/dL (1489.86 nmol/L)	—	—	<1.8 µg/dL (<50 nmol/L)
Two-day HDDST (baseline cortisol→ posttest cortisol)	57.12 → 45.5 µg/dL (1576.89 → 1255.35 nmol/L); <50% suppression	—	—	>50% suppression expected in Cushing disease

Postoperative blood pressure readings were recorded while the patient was not on any antihypertensive therapy.

Abbreviations: HDDST, high-dose dexamethasone suppression test; LDDST, low-dose dexamethasone suppression test; ONDST, overnight dexamethasone suppression test.

**Figure 2.**
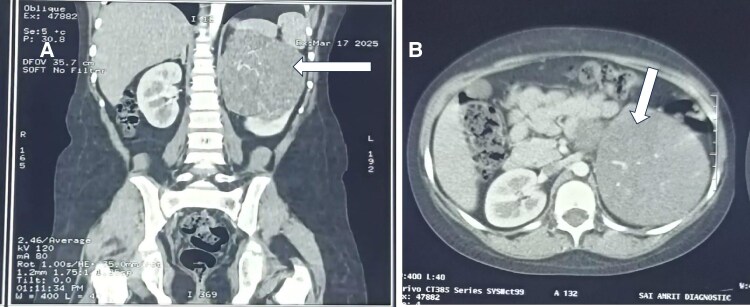
Contrast-enhanced computed tomography (CECT) abdomen demonstrating Wilms tumor. (A) Coronal section and (B) axial section reveals a well-defined large, solid, lobulated, heterodense, poorly enhancing, endo- and exophytic mass lesion measuring 11.6 × 10.2 × 10.7 cm in upper and mid-portions of the left kidney with no internal calcification or hemorrhage without involving the adrenal gland suggestive of Wilms tumor.

Magnetic resonance imaging (MRI) of the brain (contrast-enhanced) was performed to rule out pituitary Cushing disease as part of the standard evaluation for ACTH-dependent hypercortisolism. Ultrasonography (USG) of the abdomen and pelvis showed a large, well-defined, predominantly solid heterogeneous mass (∼11 × 10 × 10 cm) arising from the upper and middle regions of the left kidney, with preserved adrenal gland and no retroperitoneal lymphadenopathy or free fluid, suggestive of Wilms tumor (nephroblastoma). Contrast-enhanced computed tomography (CECT) of the abdomen was performed for detailed anatomical and vascular delineation. It revealed a large, solid, lobulated, heterodense, poorly enhancing, endophytic, and exophytic mass (11.6 × 10.2 × 10.7 cm) in the upper and middle regions of the left kidney, without internal calcification or hemorrhage. There was no evidence of adrenal hyperplasia on abdominal imaging. The overall findings were consistent with Wilms tumor (Figure 2A and 2B). High-resolution computed tomography (HRCT) of the thorax was obtained in the same setting to exclude intrathoracic ACTH-secreting lesions.

## Treatment

Hypertension was managed with oral amlodipine 5 mg twice daily (0.3 mg/kg/day) and hypokalemia was corrected with oral potassium supplements before surgery. She underwent left open radical nephrectomy with partial left adrenalectomy with perioperative stress dose of hydrocortisone. Surgery was uneventful. Hydrocortisone was continued postoperatively to prevent adrenal crisis because of hypothalamo-pituitary-adrenal (HPA) axis suppression in view of prolonged endogenous exposure to cortisol.

## Outcome and Follow-up

Postoperatively, the patient's blood pressure normalized, and antihypertensive therapy was tapered and discontinued. Serum ACTH declined promptly, measuring 9.37 pg/mL (2.06 pmol/L) (reference 7.2-63.6 pg/mL [1.59-14.01 pmol/L]) on the first postoperative day and 5.50 pg/mL (1.21 pmol/L) by the end of the first month (Table 1).

**Figure 3.**
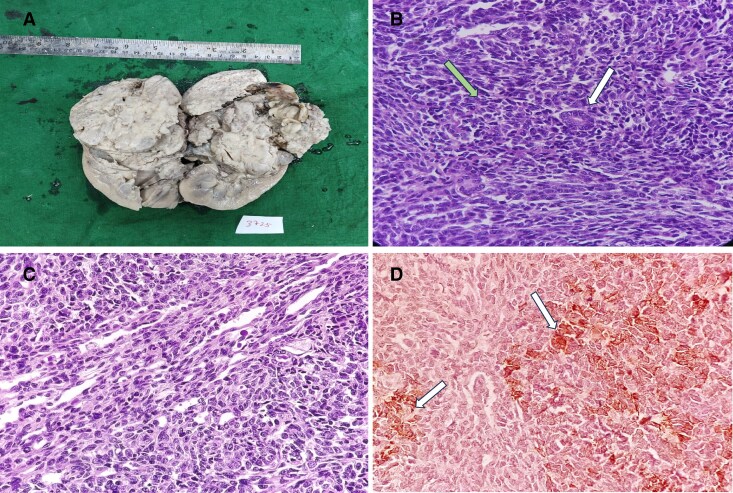
Pathological features of an ACTH-secreting Wilms tumor in a child with ectopic Cushing syndrome. (A) Gross image of the bisected nephrectomy specimen showing a well-circumscribed, lobulated mass replacing the renal parenchyma. (B) Histopathology of tumor stained with hematoxylin and eosin (H&E) highlighting the epithelial (white arrow) and blastemal (green arrow) components of the tumor. (C) H&E staining showing mesenchymal component of the tumor with stromal differentiation and spindle-shaped tumor cells. (D) ACTH immunohistochemistry showing patchy cytoplasmic positivity (arrows) in tumor cells, confirming ectopic ACTH production.

Gross examination showed a well-circumscribed lobulated mass replacing renal parenchyma (Fig. 3A). Histopathology demonstrated blastemal, stromal, and epithelial components consistent with Wilms tumor with favorable (nonanaplastic) histology as per the Children's Oncology Group classification for renal tumors (Fig. 3B–3C) [12]. There was focal tumor disruption with perinephric soft-tissue extension, whereas renal sinus and vascular margins were free of tumor and lymph nodes were negative. Based on these findings, the tumor was categorized as stage III (local) disease. Initial ACTH immunohistochemistry (IHC) was negative; repeat IHC on another block revealed focal cytoplasmic positivity across epithelial, blastemal, and stromal elements without predilection for any specific histologic pattern (Fig. 3D). However, the tumor was not studied histologically for neuroendocrine differentiation, and immunohistochemistry for neuroendocrine markers was not performed. These findings confirmed ECS secondary to a paraneoplastic ACTH-secreting Wilms tumor. The patient was started on DD4A chemotherapy (vincristine, dactinomycin, doxorubicin) with adjuvant radiotherapy [13].

## Discussion

Ectopic Cushing syndrome is a rare cause of ACTH-dependent hypercortisolism in children, and its association with Wilms tumor is exceedingly uncommon. Although Wilms tumor is the most frequent renal malignancy in childhood, paraneoplastic endocrine syndromes are rare, and ACTH-secreting variants are exceptionally so. One of the earliest cases was reported by Cummins and Cohen in 1974, where ACTH secretion was confirmed immunohistochemically [4]. A recent systematic review identified at least 8 cases of Wilms tumors associated with EAS [3]. As these tumors are usually large and easily localized on imaging, diagnosis and management are less challenging than in occult ectopic sources. Collectively, previous reports and the current case underscore the diagnostic and therapeutic challenges in recognizing and managing this rare entity (Table 2).

**Table 2.** Summary of reported cases of ectopic ACTH-secreting Wilms tumor

Author (year)	Age (y)/sex	Clinical features	ACTH	Morning serum cortisol	Imaging findings	ACTH IHC	Treatment	Outcome
Cummins & Cohen (1974) [4]	4.5/M	Cushingoid features, hypertension	880 pg/mL (193.6 pmol/L)	48 ug/dL (1316 nmol/L)	Left renal mass	Positive	Nephrectomy + chemotherapy + radiotherapy	Clinical remission
Hashimoto et al (1980) [5]	5/F	Cushingoid features, hypertension	327 pg/mL (72 pmol/L)	63 ug/dL (1734 nmol/L)	Wilms tumor	ACTH and CRF-like peptides detected	Nephrectomy + chemotherapy	Biochemical remission
Pombo et al (1982) [6]	7.5/F	Cushingoid features, hypertension	1060 pg/mL (233 pmol/L)	33 ug/dL (910 nmol/L)	Right renal mass	Not reported	Ureteronephrectomy + chemotherapy	Resolution of symptoms
Chi DH et al (1988) [7]	4 y 8 m/F	Weight gain, hypertension, moon facies	174.7 pg/mL (38.4 pmol/L)	49 ug/dL (1362 nmol/L)	Right renal mass	Positive	Nephrectomy	Resolution
Hsiao et al (1991) [8]	7/F	Cushingoid facies, HTN, hematuria	Extremely high	Elevated	Clear-cell sarcoma of kidney (Wilms tumor variant)	Positive	Nephrectomy + chemotherapy + radiotherapy	Remission
Thomas et al(1998) [9]	2 years, 8 months/M	Cushingoid facies, weight gain, hypertension	93 pg/mL (20.5 pmol/L)	32.3 ug/dL (891.16 nmol/L)	Left renal mass compressing kidney, initial suspicion of adrenocortical tumor	Not reported	Left nephroureterectomy + adrenalectomy + chemotherapy(DD4A regimen)+flank irradiation	Complete remission
Faizan M et al (2017)	2.4/M	Cushingoid features, hirsutism, hypertension	131 pg/mL (29 pmol/L)	56 ug/dL (1540 nmol/L)	Right renal mass	Not reported	Right nephrectomy + chemotherapy	Clinical and biochemical remission
Lim et al (2019) [11]	9/M	Hyperphagia, rapid weight gain, moon face	41.8 pg/mL (9.2 pmol/L)	34.5 ug/dL (952 nmol/L)	Left renal mass with no adrenal involvement	Negative	Nephrectomy + partial adrenalectomy	Resolution of symptoms
Current Case (2025)	8/F	Facial fullness, weight gain, hyperpigmentation	650 pg/mL (143.13 pmol/L)	63.06 ug/dL (1740.17 nmol/L)	Large heterogeneous left renal mass; stage III Wilms tumor	Negative initially; focal positive later	Nephrectomy + DD4A chemotherapy + radiotherapy	ACTH declined; on hydrocortisone replacement

Abbreviations: DD4A, dactinomycin, doxorubicin, vincristine chemotherapy protocol; IHC, immunohistochemistry.

Unlike adults with ECS, who often show an insidious course, pediatric patients tend to exhibit acute and florid hypercortisolism, characterized by rapid weight gain, growth failure, hypertension, and hypokalemia. In our patient, rapidly progressive cushingoid features, markedly elevated ACTH, and normal pituitary imaging prompted evaluation for an ectopic source, leading to identification of a renal mass. Thus, in pediatric CS, rapid onset and severe presentation should raise suspicion for ectopic ACTH secretion.

Surgical resection remains the mainstay of treatment for both Wilms tumor and paraneoplastic ACTH production. In accordance with pediatric oncology and endocrinology guidelines, tumor excision ensures oncologic control and normalization of hypercortisolemia. Our patient underwent left radical nephrectomy, followed by rapid normalization of blood pressure and ACTH decline (from 650 pg/mL preoperatively to 5.5 pg/mL by postoperative day 30), confirming the tumor as the source of ACTH.

Initial ACTH IHC on the tumor specimen was negative but repeat staining on a different block demonstrated focal positivity. This discrepancy can be attributed to tumor heterogeneity, where ACTH expression is often focal rather than diffuse [14]. Other factors—such as sampling variation, differences in tissue fixation, and technical limitations—may also contribute to false-negative results [14]. The mechanism underlying EAS in Wilms tumor remains speculative. A prior report described a Wilms tumor with extensive neuroendocrine differentiation, supported by histochemical, immunohistochemical, and ultrastructural evidence (Czernobilsky et al, 1989). More than 90% of blastemal cells in that case stained positively with Grimelius stain, and additional studies demonstrated neuron-specific enolase expression, suggesting the existence of a neuroendocrine variant within the histologic spectrum of Wilms tumor. Such aberrant neuroendocrine differentiation may explain the capacity of Wilms tumor cells to produce ACTH in rare cases like the present one [15]. Although ACTH IHC can aid diagnosis, it is not essential for confirming EAS. In this case, the complete clinical and biochemical remission following tumor resection provides compelling evidence that the Wilms tumor was the source of ACTH secretion. Moreover, it is noteworthy that some isolated corticotropin-releasing hormone-secreting tumors may be negative for ACTH immunostaining yet still present with ECS due to secondary pituitary corticotroph activation [16].

EAS has also been described in several nonneuroendocrine tumors, including pheochromocytoma, medullary thyroid carcinoma, ovarian tumors, and hepatocellular carcinoma [17-20]. These associations emphasize that ectopic ACTH production is not only confined to classical neuroendocrine tumors but may occur in diverse malignancies through aberrant gene expression.

Another critical aspect of management is addressing HPA axis suppression.

Chronic hypercortisolism can result in prolonged adrenal insufficiency after tumor resection [21]. In our patient, perioperative stress-dose hydrocortisone was given empirically, followed by physiological replacement, consistent with Endocrine Society guidelines recommending continued glucocorticoid therapy and periodic reassessment of adrenal recovery [22].

Although the diagnosis and outcome were conclusive, certain limitations merit mention. Screening for other potential neuroendocrine tumors using markers such as chromogranin A or 24-hour urinary metanephrines was indicated but not performed because no additional lesions were detected on imaging. Genetic evaluation for Wilms tumor syndromes (eg, *WT1* mutations, WAGR, Denys-Drash) was also not conducted, which may have implications for long-term surveillance and familial counseling [23].

In conclusion, this case underscores the need to consider ECS in ACTH-dependent hypercortisolism when pituitary and thoracic imaging are unremarkable. It highlights the diagnostic challenges of focal hormone expression, the value of repeat tissue analysis when immunostaining is inconclusive, and the importance of surgical resection for both tumor and endocrine control. Finally, vigilant perioperative glucocorticoid management and long-term adrenal monitoring are essential. Early recognition and a multidisciplinary approach remain crucial for optimizing outcomes in this rare and challenging disorder.

## Learning Points

- ECS, though rare in children, should be considered in ACTH-dependent hypercortisolism when pituitary imaging is normal and symptoms are severe or rapidly progressive.
- Wilms tumor, though common in pediatrics, is an uncommon source of ectopic ACTH production and may present with paraneoplastic hypercortisolism.
- In some cases, the ectopic source may be clinically or radiologically apparent, reducing the need for extensive localization studies.
- Negative ACTH immunostaining does not exclude ectopic hormone secretion; repeat staining of additional tumor sections may reveal focal expression.
- Definitive management requires timely tumor excision with perioperative glucocorticoid coverage and close follow-up for HPA axis recovery.

## Data Availability

Original data generated and analyzed during this study are included in this published article.

## References

[1] Tatsi C, Kamilaris C, Keil M, et al Paediatric Cushing syndrome: a prospective, multisite cohort study. Lancet Child Adolesc Health. 2024;8(1):123‐134. 10.1016/S2352-4642(23)00264-X PMC11245730 38097317

[2] Batista DL, Riar J, Keil MF, Stratakis CA. Pediatric Cushing's disease: diagnostic and therapeutic aspects. Pituitary. 2015;18(2):172‐178.

[3] Yami Channaiah C, Karlekar M, Sarathi V, et al Paediatric and adolescent ectopic Cushing's syndrome: systematic review. Eur J Endocrinol. 2023;189(4):S75‐S87. 37801647 10.1093/ejendo/lvad133

[4] Cummins GE, Cohen D. Cushing's syndrome secondary to ACTH-secreting Wilms' tumor. J Pediatr Surg. 1974;9(4):535‐539. 4367732 10.1016/s0022-3468(74)80021-7

[5] Hashimoto K, Takahara J, Ogawa N, et al Adrenocorticotropin, β-lipotropin, β-endorphin, and corticotropin-releasing factor-like activity in an adrenocorticotropin-producing nephroblastoma. J Clin Endocrinol Metab. 1980;50(3):461‐465. 6244320 10.1210/jcem-50-3-461

[6] Pombo M, Alvez F, Varela-Clives R, et al Ectopic production of ACTH by Wilms' tumor. Horm Res. 1982;16(3):160‐163. 6286443 10.1159/000179497

[7] Chi DH, Han SB, Woo YJ, Hwang TJ. A case of ectopic ACTH syndrome caused by Wilms' tumor. J Korean Pediatr Soc. 1988;31(8):1071‐1078.

[8] Hsiao JC, Yang CP, Lin CJ, Chuen H. Ectopic ACTH syndrome due to clear-cell sarcoma of the kidney. Child Nephrol Urol. 1991;11(2):103‐106. 1661639

[9] Thomas RJ, Sen S, Zachariah N, et al Wilms' tumor presenting as Cushing's syndrome. Pediatr Surg Int. 1998;13(4):293‐294. 9553194 10.1007/s003830050321

[10] Faizan M, Manzoor J, Saleem M, Anwar S, Mehmood Q, Hameed A, Ali AS. Paraneoplastic Cushing Syndrome Due To Wilm's Tumor. J Coll Physicians Surg Pak. 2017;27(5):313‐315. 28599697

[11] Lim YY, Sng AA, Ho CW-W, et al A case of Cushing syndrome in a Wilms' tumour. Horm Res Paediatr. 2019;92(Suppl 1):P3‐P1. [Poster ESPE 2019].

[12] Davidoff AM . Wilms tumor. Curr Opin Pediatr. 2009;21(3):357‐364. 19417665 10.1097/MOP.0b013e32832b323a PMC2908383

[13] Dome JS, Fernandez CV, Mullen EA, et al Children's Oncology Group's 2013 blueprint for research: renal tumors. Pediatr Blood Cancer. 2013;60(6):994‐1000. 23255438 10.1002/pbc.24419 PMC4127041

[14] Clarke TR, Haidar M, Simpson DJ, et al Ectopic ACTH production by adrenal tumors: diagnostic challenges and immunohistochemistry pitfalls. Endocr Pathol. 2011;22(3):142‐147.

[15] Czernobilsky B, Kessler E, Hertzanu Y. Neuroendocrine differentiation in Wilms' tumor: a histochemical, immunohistochemical and ultrastructural study. Cancer. 1989;63(2):280‐285.2910432

[16] Wang J, Zhang G. Paraneoplastic Cushing syndrome because of corticotrophin-releasing hormone–secreting Wilms' tumor. J Pediatr Surg. 2008;43(11):2099‐2101. 18970948 10.1016/j.jpedsurg.2008.07.014

[17] Iwasa K, Naka G, Ohno Y, et al Cushing's syndrome due to ectopic ACTH secretion by pheochromocytoma. Intern Med. 1992;31(4):479‐483.

[18] Barlier A, Pellegrini I, Gunz G, et al Ectopic ACTH syndrome in medullary thyroid carcinoma. J Clin Endocrinol Metab. 1993;77(6):1725‐1728.

[19] Griffing GT, Melby JC, Orth DN. Ectopic production of ACTH by an ovarian tumor. Arch Intern Med. 1977;137(6):881‐884.

[20] Nomura K, Kimura H, Tanaka S, et al Cushing's syndrome due to ectopic ACTH production by hepatocellular carcinoma. Cancer. 1986;57(10):1964‐1968.

[21] Valassi E, Biller BMK, Swearingen B, et al Delayed remission after transsphenoidal surgery in Cushing's disease: the need for long-term follow-up. J Clin Endocrinol Metab. 2010;95(2):601‐610. 20080848 10.1210/jc.2009-1672 PMC2840864

[22] Nieman LK . Glucocorticoid replacement therapy: a pharmacologic perspective. Endocrinol Metab Clin North Am. 2011;40(2):273‐283.

[23] Dome JS, Perlman EJ, Graf N. Risk stratification for Wilms tumor: current approach and future directions. Am Soc Clin Oncol Educ Book. 2014;34:e194‐e198. 10.14694/EdBook_AM.2014.34.215 24857079

